# A case report of acute intermittent porphyria presenting with reversible cerebral vasoconstriction syndrome

**DOI:** 10.1097/MD.0000000000041526

**Published:** 2025-02-21

**Authors:** Wei Rao, Lingjuan Li, Lingfeng Wu, Wen Chai, Jie Li

**Affiliations:** a Department of Neurology, Jiangxi Provincial People’s Hospital, The First Affiliated Hospital of Nanchang Medical College, Nanchang, Jiangxi Province, China.

**Keywords:** acute intermittent porphyria, HMBS, PBG, reversible cerebral vasoconstriction syndrome

## Abstract

**Rationale:**

Acute intermittent porphyria (AIP), an autosomal dominant genetic disorder, is primarily characterized by neuropsychiatric abnormalities and intermittent abdominal pain; however, it is extremely rare for AIP to present with reversible cerebral vasoconstriction syndrome (RCVS).

**Patient concerns:**

Herein, we report a case of AIP presenting with RCVS in a patient admitted to our hospital.

**Diagnoses:**

We present a case of a 16-year-old female patient with clinical manifestations including abdominal pain, seizures, headache, and peripheral nerve lesions. Laboratory examination revealed small-cell hypochromic anemia, hyponatremia, hypochloremia, and a positive result of the urine porphobilinogen sun exposure test. The peripheral nerve injury was detected via electromyography. A left occipital epileptic discharge was observed on an electroencephalography. Brain magnetic resonance imaging (MRI) revealed abnormal signals in the bilateral temporal, parietal, and occipital lobes, and stenosis in multiple intracranial vasculatures. The patient was found to carry a mutation site in the hydroxymethylbilane synthase (HMBS) gene, and her father was heterozygous.

**Interventions:**

After treatment with high glucose, heme arginine, antiepileptic therapy, correction of electrolyte disorders, and nutritional support, the patient’s symptoms were improved considerably.

**Outcomes:**

Repeat brain MRI showed that the lesion disappeared and the intracranial vasculature returned to normal. The electromyography reexamination results also demonstrated that the peripheral nerve injury was relieved.

**Lessons:**

RCVS may occur in AIP patients, which is featured with abdominal pain, neuropsychiatric symptoms, and stenosis in multiple intracranial vasculatures. Urine porphobilinogen sun exposure test, brain MRI, and genetic examination can aid in a definitive diagnosis. A high glucose diet, heme arginine, and symptomatic treatment are effective in treating AIP presenting with RCVS.

## 1. Introduction

Acute intermittent porphyria (AIP) is an autosomal dominant genetic disorder caused by mutations in the hydroxymethylbenzene synthase (HMBS) gene, and lack of enzyme activity leads to porphyrin or its precursors, including porphobilinogen (PBG) and delta-aminolevulinic acid (ALA), deposition in various locations.^[1]^ They are neurotoxic, and can result in a variety of clinical manifestations like abdominal pain, neuropsychiatric abnormalities, brown-red urine, and other symptoms.^[2]^ The global epidemiological incidence of AIP is 1 to 9 per million, and the annual incidence of symptomatic AIP in Europe is 0.13 per million.^[3]^ Up to now, there is no epidemiological data available in China. Factors such as fatigue, hunger, stress, sex hormones, drugs, and alcohol can induce or aggravate AIP.^[4]^ Reversible cerebral vasoconstriction syndrome (RCVS) is a group of clinical manifestations characterized by lightning-like headaches, with or without focal signs in the nervous system, and neuroimaging reveals multifocal, segmental, and reversible cerebral vasoconstriction.^[5]^ Although studies have shown that the prognosis of RCVS is generally favorable, seizure or stroke may happen occasionally.^[6]^ AIP presenting with RCVS is rare. In this study, we report a case of AIP patient comorbidity with RCVS and review the relevant literature.

## 2. Case presentation

A 16-year-old female patient was admitted to our hospital with paroxysmal abdominal pain for 4 months and seizures for 4 days. In January 2022, the patient underwent abdominal pain, especially around the middle of the abdomen and umbilicus, which spontaneously disappeared after more than 10 days. Subsequently, she suffered from abdominal pain again, without any fixed position but mainly in the right lower abdomen, for which she was diagnosed with “acute appendicitis.” Then she was operated with a laparoscopic appendicitis, but her abdominal pain persisted after the operation. On April 30, she developed severe headache with unbearable pain, mostly located in the bilateral frontal, parietal, and occipital areas, accompanied with worsening abdominal pain and limb weakness. Additionally, focal seizures affecting the right side of her face progressed to tonic-clonic seizures for 5 times, lasting approximately a minute in each session. Prior to this onset, the patient was treated with starvation therapy for weight loss for 6 months. The patient had no similar medical family history, except that her father had a history of epileptic seizures but did not sought any medical attention.

**Figure 1.**
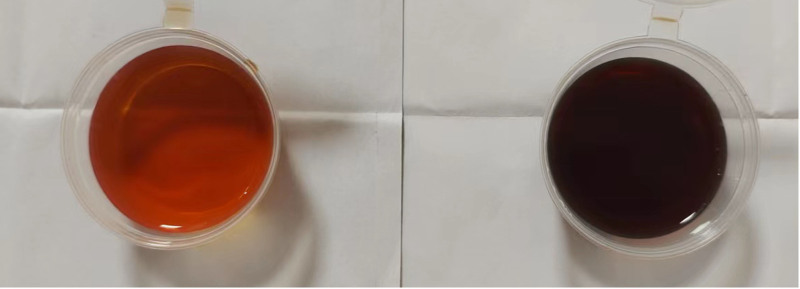
Change in urine color from yellow to brown-red after sun exposure during acute attack phase.

On admission to our hospital, her vital signs included a temperature of 36.3°C, a heart rate of 124 beats per minute, and a blood pressure of 154/110 mm Hg. Physical examination found a pain in the lower abdomen, with reduced tendon reflexes and a grade 3 extremity muscle strength. No other neuropsychiatric symptom was noted. Laboratory examinations indicated small-cell hypochromic anemia, hyponatremia, hypochloremia, and a positive result of urinary porphobilinogen (PBG) sun exposure (Fig. 1). The lactate levels in her blood were normal. Laboratory test results were negative for antibodies against autoimmune encephalitis, TPHA, RPR, HIV, ANA, anticardiolipin, lupus anticoagulant, and 2GP1. High-throughput metagenomic sequencing on pathogenic microorganisms yielded negative results.

**Figure 2.**
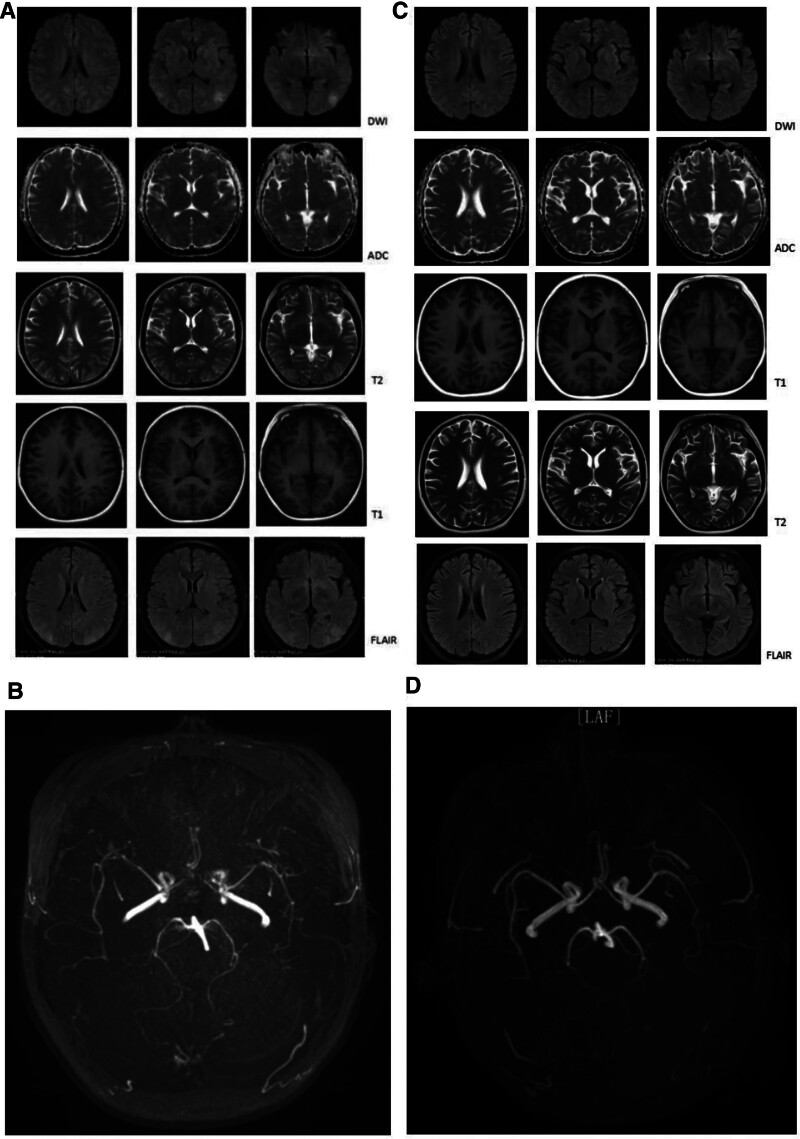
(A) Brain MRI showing hypointense signals on T1 and ADC scan in bilateral temporal, parietal, and occipital lobes while revealing hyperintense signals on T2 and FLAIR during acute attack phase. (B) MRA showing multiple segmental stenosis in bilateral anterior cerebral artery, middle cerebral artery, and posterior cerebral artery during acute attack phase. (C) Brain MRI showing disappearance of lesions in bilateral temporal, parietal, and occipital lobes during the remission period. (D) MRA revealing normal intracranial vessels during the remission period. ADC = apparent diffusion coefficient, FLAIR = fluid attenuated inversion recovery, MRA = magnetic resonance angiography, MRI = magnetic resonance imaging.

The electrocardiography revealed a sinus tachycardia in the patient. Electromyography showed that the motion amplitude of the left median and bilateral common peroneal nerves was decreased, while the latency of the bilateral common peroneal nerve was increased. In addition, it demonstrated a decrease in the F-wave occurrence rate of the bilateral median and posterior tibial nerves. An epileptic discharge in the left occipital lobe was captured by the electroencephalography (EEG). Her brain magnetic resonance imaging (MRI) detected hypointense signals on T1 and apparent diffusion coefficient scans in the bilateral temporal, parietal, and occipital lobes, whereas hyperintense signals were identified on T2 and fluid attenuated inversion recovery images (Fig. 2A). Magnetic resonance angiography (MRA) suggested multiple segmental stenoses in bilateral anterior cerebral artery, middle cerebral artery, and posterior cerebral artery (Fig. 2B).

**Figure 3.**
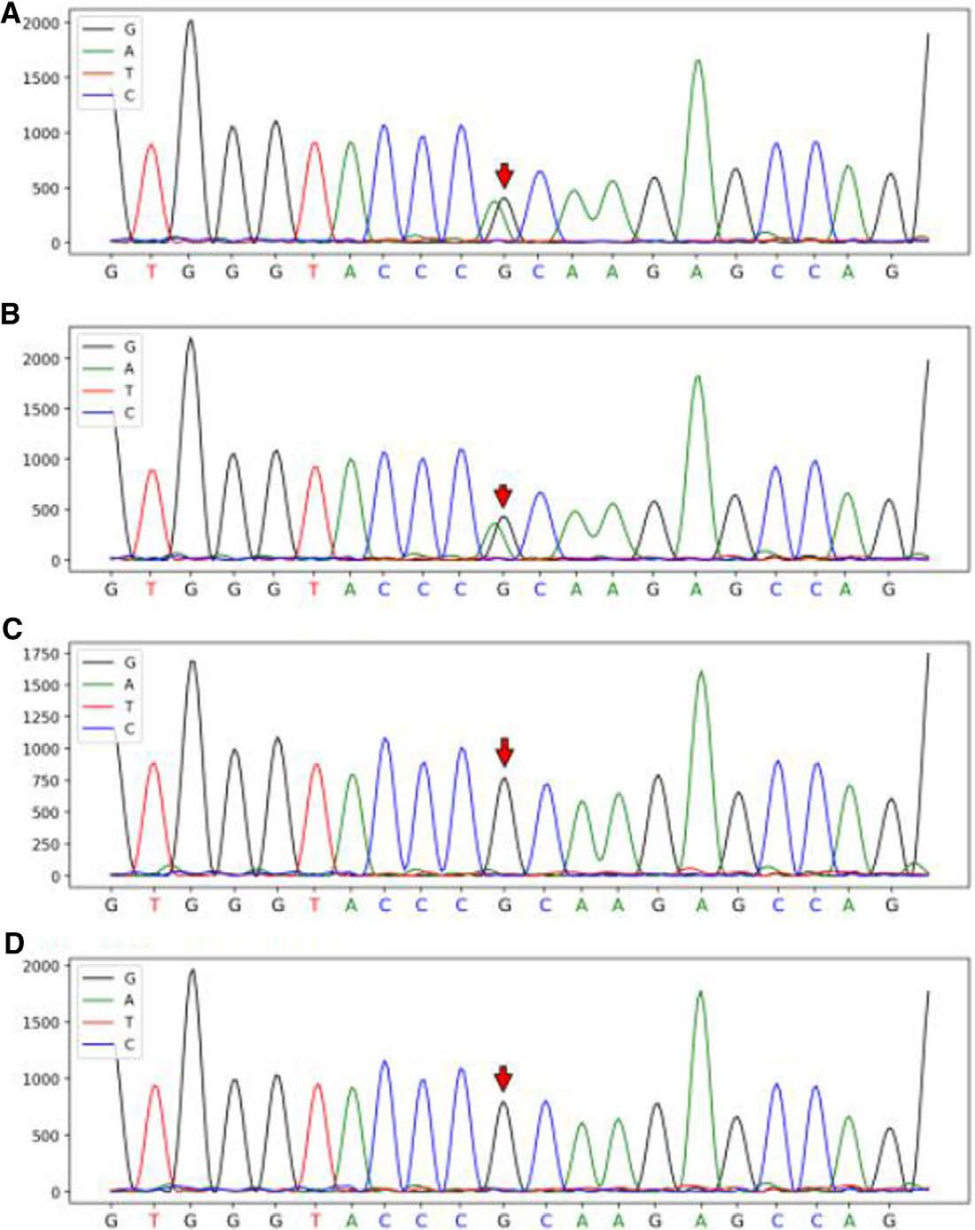
Sanger sequencing pedigree verification diagram. (A) HMBS gene mutation (c.77G>A) in patient; (B) HMBS gene mutation (c.77G>A) in patient’s father; (C) wild type in patient’s mother; (D) wild type in patient’s brother. HMBS = hydroxymethylbilane synthase.

Porphyria gene sequencing manifested that the patient carried a heterozygous variation in the HMBS gene: HMBS chr11:118959008 NM_000190. Exon2 c.77G>A (p. R26H) (Fig. 3). R26H mutation has been previously reported to result in an inactive enzyme, which is unable to produce the HMB product.^[7]^ We also predicted the pathogenicity of this missense mutation with 4 methods. The SIFT software predicted it to be deleterious, Polyphen2 software judged it to be probably damaged, and Mutation Taster software deemed it to be automatically disease causing. According to the interpretation guide for American College of Medical Genetics gene mutations, R26H mutation was graded as likely pathogenic. Besides, the patient’s father also carried a heterozygous variation in HMBS, and both of her mother and brother were wild (Fig. 3).

The patient’s was treated with high glucose, heme arginine (Normosang) (3 mg/kg for 4 consecutive days), corrected electrolyte disorders, and antiepileptic therapy. Then her symptoms were relieved significantly, with alleviated headache and abdominal pain, stopped epileptic seizure, improved limb weakness, and restored electrolyte levels. On June 22, brain MRI showed that the lesions in bilateral temporal, parietal, and occipital lobe had disappeared (Fig. 2C), and MRA showed the intracranial vessels returned to normal (Fig. 2D). Additionally, the electromyography (EMG) showed decreased motor amplitude and prolonged latency of the left common peroneal nerve.

## 3. Discussion

AIP is an autosomal dominant genetic disorder caused by mutations in HMBS, which is mainly characterized by abdominal pain, neuropsychiatric abnormalities, brown-red urine, and other symptoms.^[8–10]^ AIP that affects the digestive system may cause abdominal pain, nausea, and vomiting, because of the neurotoxicity of porphyrin precursors in the liver and the stimulation of gastrointestinal smooth muscle. When it involves in the central nervous system, it may manifest as epilepsy, psychiatric symptoms, or damage to the cerebral cortex. The mechanism of neurotoxicity of porphyrin precursors may be related to oxidative damage, mitochondrial metabolic disorders, and dysfunction of gamma-aminobutyric acid. The psychiatric symptoms of AIP may include anxiety, depression, insomnia, delusions, hallucinations, and changes in consciousness, which are mainly attributed to the direct toxicity of ALA and the main neurotransmitter that related to ALA.^[8,11]^ If the peripheral nervous system is implicated in, limb weakness, numbness, and peripheral nerve damage may occur, as indicated by electromyography. Involvement of the autonomic nervous system may lead to tachycardia and hypertension. Involvement of the blood system may lead to anemia. Hyponatremia and hypochloremia can arise from hypothalamic involvement, known as abnormal antidiuretic hormone secretion.

The diagnostic criteria for AIP are as follows: typical clinical symptoms, including abdominal pain, neuropsychiatric abnormalities, and brown-red urine; laboratory examination: quantitative and qualitative increase in urine PBG and increased urine ALA;^[12]^ and genetic test: HMBS gene mutation.^[13]^ The patient exhibited symptoms, such as abdominal pain, seizures, and peripheral nerve lesions. Particular attention was paid to distinguish between AIP and mitochondrial neurogastrointestinal encephalopathy disease (MNGIE). However, the absence of ophthalmoplegia, cachexia and severe leukoencephalopathy on the brain MRI temporarily ruled out MNGIE. Urinary PBG sun exposure testing revealed changes in urine color from yellow to brown-red (Fig. 1), and the porphyria gene sequencing results revealed that the patient and her father carried a heterozygous variation in the HMBS gene: HMBS chr11:118959008 NM_000190. Exon2 c.77G>A (p. R26H). Given that the patient’s onset was caused by an incomplete penetrance of the mutation, the disease was a dominant genetic disorder; however, the patient’s father underwent only once of epileptic seizure and had no other comparable symptoms. The patient’s heterozygous mutation in the HMBS gene was considered pathogenic. AIP was diagnosed in our case because of the presence of abdominal pain, seizures, peripheral nerve lesions, small-cell hypochromic anemia, hyponatremia, and hypochloremia, as well as positive results for urinary PBG tests and genetic analysis. Therefore, AIP should be considered in patients with unexplained abdominal pain, mental symptoms, or clinical manifestations of neurological symptoms.

Currently, there is no radical cure for AIP. Prompt implementation of heme preparations and high-sugar treatments are the mainstay of acute attack treatment. By inhibiting the peroxisome proliferator-activated receptor γ coactivator-1α, high glucose levels can downregulate delta-aminolevulinic acid synthase 1 (ALAS1), a heme biosynthesis pathway enzyme, thereby reducing the excretion of porphyrin precursors. Therefore, a high-sugar diet helps to alleviate the acute onset of AIP symptoms. Heme preparations such as heme arginine and chlormethemoglobin can inhibit ALAS1 activity, thereby reducing ALA and PBG synthesis, which can rapidly alleviate the symptoms.^[1,13]^ The patient’s symptoms significantly improved after treatment with high glucose, arginine heme, corrected electrolyte disorders, and antiepileptic therapy. The electrolyte level returned to normal, bilateral lesions in the temporal, parietal, and occipital lobes disappeared, and cerebrovascular stenosis vanished according to a repeat brain MRI scan. Furthermore, the EMG examination results significantly improved. Therefore, it is indispensable to maintain body fluid balance, to correct hydroelectrolyte disorders, to relieve abdominal pain, to control seizures, and to relieve the mental and neurological symptoms, during acute AIP attacks. Although most acute AIP symptoms are reversible and recurrent, it can be fatal in severe cases, and patient prognosis can be considerably improved through effective and timely diagnosis and treatment.

Upon admission, brain MRA showed numerous segmental stenoses in bilateral anterior, middle, and posterior cerebral arteries, along with severe headache symptoms and high blood pressure. Porphyrin and its precursors may deposit in the brain tissue, thus leading to cerebral vasospasm and cerebral cortex damage. The patient was diagnosed with RCVS because cerebral vasospasm was alleviated during the period of remission, with the disappearance of cortical lesions and reversible vasoconstriction. Therefore, AIP may be a cause of RCVS, which is a group of syndromes with clinical manifestations of multifocal, segmental, reversible cerebral vasoconstriction and severe headache (lightning-like headache), with or without focal neurological signs. The pathological basis is the reversible contraction of cerebral blood vessels.^[5]^ However, the underlying mechanisms have not yet been elucidated. It may be attributed to the dysregulation of cerebral vascular tone caused by internal or external factors or to the enhanced sympathetic stimulation.^[14]^ Possible contributors include postpartum conditions, vasoactive drug usage (such as triptans and selective serotonin reuptake inhibitors), catecholamine-secreting tumors, immunosuppressants or blood products, hypercalcemia, porphyria, and cerebral venous thrombosis.^[15]^ The development of RCVS in this patient was thought to be caused by the accumulation of porphyrin or its precursors in the tissues, which causes dysregulation of cerebral vascular tone, extensive cerebral vasospasm, multiple vascular stenoses, elevated blood pressure, excruciating headaches, and numerous abnormal signals in the bilateral cerebral cortex. Fortunately, the imaging findings were reversible.

AIP combination with RCVS is rare and the diagnosis is complex. The diagnosis of AIP was clear, with MRA indicating multiple segmental stenoses. Considering their correlation, there was an overlap in the course and duration of AIP and RCVS. Previous studies have reported that porphyria is a cause of RCVS,^[15]^ whereas AIP presenting as RCVS is rare and has not been reported domestically or internationally. At present, the literature suggests that the neurotoxicity of porphyrin precursors and the resulting damage to endothelial cells can cause vasospasm and reversible vasoconstriction. In addition, porphyrin metabolism disorders may lead to dysfunction of NO synthase, resulting in a decrease in NO synthesis that causes vasodilation, which contributes to vasospasm.^[16]^ During an acute attack of AIP, the head MRA manifestations are reversible, and the abnormal signals on the head MRI are reversible. This may be due to the vascular edema caused by vasospasm accompanied by RCVS. Reversible manifestations on head MRA hold significant diagnostic value for AIP. However, its exact pathogenesis remains unclear and requires further clinical data.

Currently, the pathological mechanism of RCVS is unclear, but impaired cerebrovascular function may be a key factor in its pathogenesis. Most RCVS may be secondary to certain triggering factors, mainly related to the use of vasoactive substances postpartum or during pregnancy.^[17,18]^ Disruption of the blood-brain barrier may also be a factor in the pathogenesis of RCVS. Some studies suggest that the occurrence of RCVS overlaps with the pathology of reversible posterior leukoencephalopathy (PRES). RCVS can cause brain edema and reversible changes, as evident from head MRI and MRA. Moreover, the incidence of reversible abnormal MRI and MRA images in PRES is similarly high.^[19]^ Endothelial cell dysfunction may also be involved in RCVS development. Currently, there are certain literature reports on AIP manifesting as PRES, but there are no relevant studies on AIP manifesting as RCVS at home or abroad.

## Author contributions

**Investigation:** Wei Rao, Lingfeng Wu, Wen Chai.

**Resources:** Lingfeng Wu, Wen Chai, Jie Li.

**Methodology**: Wei Rao, Lingjuan Li.

**Writing – original draft**: Wei Rao, Lingjuan Li.

**Writing – review &editing**: Wei Rao, Lingjuan Li, Lingfeng Wu, Wen Chai, Jie Li.
